# Supplementary material for: Jouvence a small nucleolar RNA required in the gut extends lifespan in Drosophila
Source: Nat Commun. 2020 Feb 20;11:987. doi: 10.1038/s41467-020-14784-1 (PMC7033134; doi:10.1038/s41467-020-14784-1)
Supplement: Supplementary file 1 — Supplementary Information [file 41467_2020_14784_MOESM1_ESM.pdf]

SUPPLEMENTARY INFORMATION

jouvence a new small nucleolar RNA required in the gut extends lifespan in Drosophila

Stéphanie Soulé[1#], Lucille Mellottée[1#], Abdelkrim Arab[1], Chongjian Chen[2], and Jean-René Martin[1*]

(1) Equipe: Imagerie Cérébrale Fonctionnelle et Comportements (ICFC),
Institut des Neurosciences Paris-Saclay (Neuro-PSI)
UMR-9197, CNRS/Université Paris Sud,
1 Avenue de la Terrasse (Bat. 32/33)
91198, Gif-sur-Yvette, France
Tel: (33) 01.69.82.41.80 / Fax: (33) 01.69.82.34.47
(1*) corresponding author: Jean-René Martin: e-mail: jean-rene.martin@inaf.cnrs-gif.fr
# : these authors contribute equally to this work.

Supplementary Table 1 (Statistic of Longevity)

Name	n	Age in days at % mortality 25%	50%	75%	90%	100%	Statistics: Condition	P-value
**in Deletion-F4**								
Control (CS)	287	34,0	40,0	44,0	50,0	56,0	Control (CS) v.s. Del (Deletion-F4)	0,000
Del (Deletion-F4)	302	28,0	34,0	36,0	44,0	58,0	Control (CS) v.s. Del+rescue-1	0,154
Del+rescue-1	173	32,0	40,0	44,0	48,0	52,0	Control (CS) v.s. rescue-1	0,000
rescue-1	259	41,0	45,0	49,0	55,0	63,0	Del (Deletion-F4) v.s. Del+rescue-1	0,000
Del (Deletion-F4)	218	30,0	32,0	36,0	36,0	38,0	Del (Deletion-F4) v.s. Del+rescue-2	0,000
Del+rescue-2	138	34,0	44,0	50,0	52,0	54,0	Del (Deletion-F4) v.s. Del+rescue-3	0,000
Del+rescue-3	170	41,0	45,0	47,0	51,0	55,0		
Del,Myo1A>UAS-jou (rescued)	104	59,0	64,0	71,0	71,0	76,0	Del,Myo1A/UAS-jou v.s. Control-1 (Del,Myo1A/Del)	0,000
Control-1 (Del,Myo1A/Del)	111	49,0	56,0	60,0	63,0	63,0	Del,Myo1A/UAS-jou v.s. Control-2 (Del,UAS-jou/Del)	0,000
Control-2 (Del,UAS-jou/Del)	112	49,0	53,0	56,0	63,0	64,0		
Del,Myo1A>UAS-sno-2	216	42,0	46,0	50,0	54,0	54,0	Del,Myo1A/UAS-sno-2 v.s. Control-1 (Del,Myo1A/Del)	0,000
Control-1 (Del,Myo1A/Del)	241	41,0	49,0	53,0	55,0	59,0	Del,Myo1A/UAS-sno-2 v.s. Control-2 (Del,UAS-sno-2/Del)	0,000
Control-2 (Del,UAS-sno-2/Del)	242	18,0	20,0	22,0	24,0	26,0		
Del,Myo1A>UAS-sno-3	235	40,0	44,0	46,0	50,0	52,0	Del,Myo1A/UAS-sno-3 v.s. Control-1 (Del,Myo1A/Del)	0,000
Control-1 (Del,Myo1A/Del)	241	41,0	49,0	53,0	55,0	59,0	Del,Myo1A/UAS-sno-3 v.s. Control-2 (Del,UAS-sno3/Del)	0,000
Control-2 (Del,UAS-sno3/Del)	231	28,0	34,0	36,0	38,0	42,0		
Del,Mex>UAS-jou (rescued)	98	55,0	63,0	67,0	71,0	71,0	Del,Mex/UAS-jou v.s. Control-1 (Del,Mex/Del)	0,000
Control-1 (Del,Mex/Del)	124	47,0	53,0	59,0	63,0	63,0	Del,Mex/UAS-jou v.s. Control-2 (Del,UAS-jou/Del)	0,000
Control-2 (Del,UAS-jou/Del)	112	49,0	53,0	56,0	63,0	64,0		
Del,Mex-GS>UAS-jou (RU)	198	56,0	58,0	62,0	64,0	70,0	Del,Mex-GS/jou (RU) v.s. Del,Mex-GS/jou (No RU)	0,000
Del,Mex-GS>UAS-jou (No RU)	212	48,0	50,0	54,0	58,0	64,0		
Del,Mex-GS>UAS-sno2 (RU)	66	29,0	33,0	37,0	39,0	43,0	Del,Mex-GS/sno2 (RU) v.s. Del,Mex-GS/sno2 (No RU)	0,000
Del,Mex-GS>UAS-sno2 (No RU)	101	34,0	36,0	38,0	40,0	46,0		
Del,Mex-GS>UAS-sno-3 (RU)	134	35,0	37,0	39,0	41,0	43,0	Del,Mex-GS-sno-3 (RU) v.s. Del,Mex-GS/sno-3 (No RU)	0,000
Del,Mex-GS>UAS-sno-3 (No RU)	70	52,0	60,0	64,0	66,0	68,0		
Del,Mex-GS>UAS-cDNA-CG6296 (RU)	220	40,0	56,0	64,0	68,0	76,0	Del,Mex-GS>UAS-cDNA-CG6296 (RU)	0,000
Del,Mex-GS>UAS-cDNA-CG6296 (No RU	215	32,0	36,0	40,0	42,0	58,0		
Del,Mex-GS>UAS-ninaD-RNAi (RU)	195	52,0	64,0	68,0	70,0	76,0	Del,Mex-GS>UAS-ninaD-RNAi (RU) v.s. (No RU)	0,000
Del,Mex-GS>UAS-ninaD-RNAi (No RU)	223	33,0	37,0	41,0	47,0	59,0		
**Over-expression**								
Myo1A>UAS-jou	170	42,0	56,0	64,0	66,0	72,0	Myo1A/UAS-jou v.s. Control-1 (Myo1A/+)	0,000
Control-1 (Myo1A/+)	203	21,0	39,0	49,0	53,0	63,0	Myo1A/UAS-jou v.s. Control-2 (UAS-jou/+)	0,000
Control-2 (UAS-jou/+)	214	35,0	49,0	57,0	63,0	69,0		
Mex>UAS-jou	167	46,0	54,0	58,0	62,0	68,0	Mex/UAS-jou v.s. Control-1 (Mex/+)	0,000
Control-1 (Mex/+)	203	32,0	44,0	50,0	58,0	62,0	Mex/UAS-jou v.s. Control-2 (UAS-jou/+)	0,000
Control-2 (UAS-jou/+)	266	23,0	43,0	49,0	55,0	61,0		
Mex-GS>UAS-jou (RU)	195	53,0	59,0	63,0	65,0	73,0	Mex-GS/UAS-jou (RU) v.s. Mex-GS/UAS-jou (No RU)	0,000
Mex-GS>UAS-jou (No RU)	199	51,0	55,0	59,0	61,0	65,0		
Mex-GS>UAS-sno2 (RU)	210	41,0	49,0	59,0	63,0	71,0	Mex-GS/UAS-sno2 (RU) v.s. Mex-GS/UAS-sno2 (No RU)	0,476
Mex-GS>UAS-sno2 (No RU)	196	43,0	49,0	59,0	61,0	73,0		
Mex-GS>UAS-sno3 (RU)	222	48,0	54,0	62,0	66,0	72,0	Mex-GS/UAS-sno3 (RU) v.s. Mex-GS/UAS-sno3 (No RU)	0,169
Mex-GS>UAS-sno3 (No RU)	204	49,0	53,0	59,0	65,0	75,0		

Supplementary Table 2 (Statistics of Stress tests)

			Age in days at % mortality					Statistics	
**Condition**	**Name**	**n**	**25%**	**50%**	**75%**	**90%**	**100%**	**Condition**	**P-value**
**Dessication (young)**	Control (CS)	100	5,0	6,0	7,0	7,0	8,0	Control (CS) v.s. Del (Deletion-F4)	0,000
	Del (Deletion-F4)	100	3,0	5,0	6,0	7,0	7,0	Control (CS) v.s. Del+rescue-1	0,056
	Del+rescue-1	120	5,0	5,0	6,0	7,0	7,0	Control (CS) v.s. rrescue-1	0,000
	rrescue-1	100	5,0	6,0	8,0	9,0	9,0	Del (Deletion-F4) v.s. Del+rescue-1	0,007
**Dessication (old)**	Control (CS)	96	6,0	8,0	9,0	10,0	11,0	Control (CS) v.s. Del (Deletion-F4)	0,024
	Del (Deletion-F4)	108	5,0	8,0	9,0	10,0	11,0	Control (CS) v.s. Del+rescue-1	0,000
	Del+rescue-1	75	9,0	10,0	11,0	12,0	12,0	Control (CS) v.s. rescue-1	0,001
	rescue-1	93	8,0	9,0	10,0	11,0	11,0	Del (Deletion-F4) v.s. Del+rescue-1	0,000
**Starvation (young)**	Control (CS)	100	48,0	48,0	60,0	60,0	72,0	Control (CS) v.s. Del (Deletion-F4)	0,000
	Del (Deletion-F4)	100	48,0	60,0	60,0	72,0	84,0	Control (CS) v.s. Del+rescue-1	0,000
	Del+rescue-1	100	48,0	48,0	60,0	72,0	84,0	Control (CS) v.s. rescue-1	0,090
	rescue-1	100	48,0	48,0	60,0	72,0	84,0	Del (Deletion-F4) v.s. Del+rescue-1	0,410
**Starvation (old)**	Control (CS)	100	36,0	36,0	48,0	60,0	60,0	Control (CS) v.s. Del (Deletion-F4)	0,707
	Del (Deletion-F4)	100	24,0	36,0	48,0	48,0	72,0	Control (CS) v.s. Del+rescue-1	0,000
	Del+rescue-1	100	36,0	48,0	48,0	60,0	72,0	Control (CS) v.s. rescue-1	0,040
	rescue-1	100	36,0	36,0	48,0	60,0	72,0	Del (Deletion-F4) v.s. Del+rescue-1	0,002
**Dessication (young)**	Del,Myo1A>UAS-jou (rescued)	100	5,0	6,0	7,0	8,0	10,0	Del,Myo1A/jou (rescued) v.s. Control-1 (Del,Myo1A/Del)	0,000
	Control-1 (Del,Myo1A/Del)	100	5,0	5,0	6,0	7,0	8,0	Del,Myo1A/jou (rescued) v.s. Control-2 (Del,UAS-jou/Del)	0,001
	Control-2 (Del,UAS-jou/Del)	169	4,0	5,0	6,0	8,0	9,0		
**Dessication (old)**	Del,Myo1A>UAS-jou (rescued)	70	7,0	9,0	10,0	11,0	12,0	Del,Myo1A/jou (rescued) v.s. Control-1 (Del,Myo1A/Del)	0,000
	Control-1 (Del,Myo1A/Del)	90	6,0	7,0	8,0	9,0	11,0	Del,Myo1A/jou (rescued) v.s. Control-2 (Del,UAS-jou/Del))	0,000
	Control-2 (Del,UAS-jou/Del))	65	5,0	7,0	9,0	10,0	11,0		
**Starvation (young)**	Del,Myo1A>UAS-jou (rescued)	180	48,0	60,0	72,0	84,0	84,0	Del,Myo1A/jou (rescued) v.s. Control-1 (Del,Myo1A/Del)	0,000
	Control-1 (Del,Myo1A/Del)	200	36,0	48,0	60,0	72,0	84,0	Del,Myo1A/jou (rescued) v.s. Control-2 (Del,UAS-jou/Del)	0,120
	Control-2 (Del,UAS-jou/Del)	52	48,0	48,0	60,0	72,0	84,0		
**Starvation (old)**	Del,Myo1A/UAS-jou (rescued)	80	48,0	60,0	72,0	84,0	84,0	Del,Myo1A/jou (rescued) v.s. Control-1 (Del,Myo1A/Del)	0,000
	Control-1 (Del,Myo1A/Del)	120	36,0	48,0	60,0	60,0	72,0	Del,Myo1A/-jou (rescued) v.s. Control-2 (Del,UAS-jou/Del)	0,000
	Control-2 (Del,UAS-jou/Del)	74	36,0	48,0	60,0	72,0	84,0		

Supplementary Experimental Procedures

Sequence of Primers used in this study.

- Drosophila 66851F = 5'-GATACGACTGAAGGTCTCACT-3'

- Drosophila 68140R = 5'-CAAGCTGATCATGCAAGAGG-3'

- snoRNAgenomic-F: 5’-GCGC*TCTAGA*GGACATGGCTTTAGTATTCC-3’

- snoRNAgenomic-R: 5’-CG*TCTAGA*ACATTGCCTCCGTACATCCA-3’

- Drosophila forward: (snoRNA-Fb) (5’-AAAGCGTTAGATATTAAACTG-3’)

- Drosophila reverse (snoRNA-Rb) (5’-TACTGTGGTTAGTACCTC-3’)

- sno-2: 67163F + Bgl2: 5’-CG AGATCT AATGTGCGTTGTCCCATGCA-3’

  67329R + Xho1: 5’-GGCCCG CTCGAG GGTTAATAGAATTCATAATTGTTC-3’

- sno-3: 66963F + Bgl2: 5’-GGGCCC AGATCT TGTTGTCCAATGCACGCTCA-3’

  67118R + Xho1: 5’-GGGCCC CTCGAG GTATGTATACCTAATTGTTTGAC-3’

- Drosophila rp49 forward primer: 5’-CACAAATGGCGCAAGCCCAAGG-3’

- Drosophila rp49 reverse primer: 5’-TTGAGAACGCAGGCGACCGTTG-3’

- Cycline E: Forward: GACGACTCGCACATTATCCA

  Reverse: CTTGTGACTAGAGGCGGGAG

Pseudouridylation (*Drosophila*) :

- PseudoU-Reverse-18S : TGCCAACATGAATGAAGGCT

- PseudoU-Reverse-28S : AAGATCGTTTCGACCCTAAG

Primer to generate the genomic ribosomal DNA fragment for sequencing:

- 18S-Forward: TCTCGAATCTATGGGTGGTG

- 18S-reverse : GGACCTCTCGGTCTAGTAAA

- 28S-Forward: CGAAACAGTTCTGACGTGCA

- 28S-Reverse: TGGGCACCGTAACATTACGT

Mouse:

Ctnnd2 : Forward: GGGAAATGATCAGCCTCAAA

Reverse: GACAGGCTGTGTGGAAACCT

EPC1 : Forward: GGGTTCAAGGTGAAGGATGA

Reverse: GGGAACCCGAAGTCTGTGTA

Human:

TEAD1 : Forward: ACCAAACCCAGGAGGAGACT

Reverse : ACCCCTTCTGCATCATTGTC

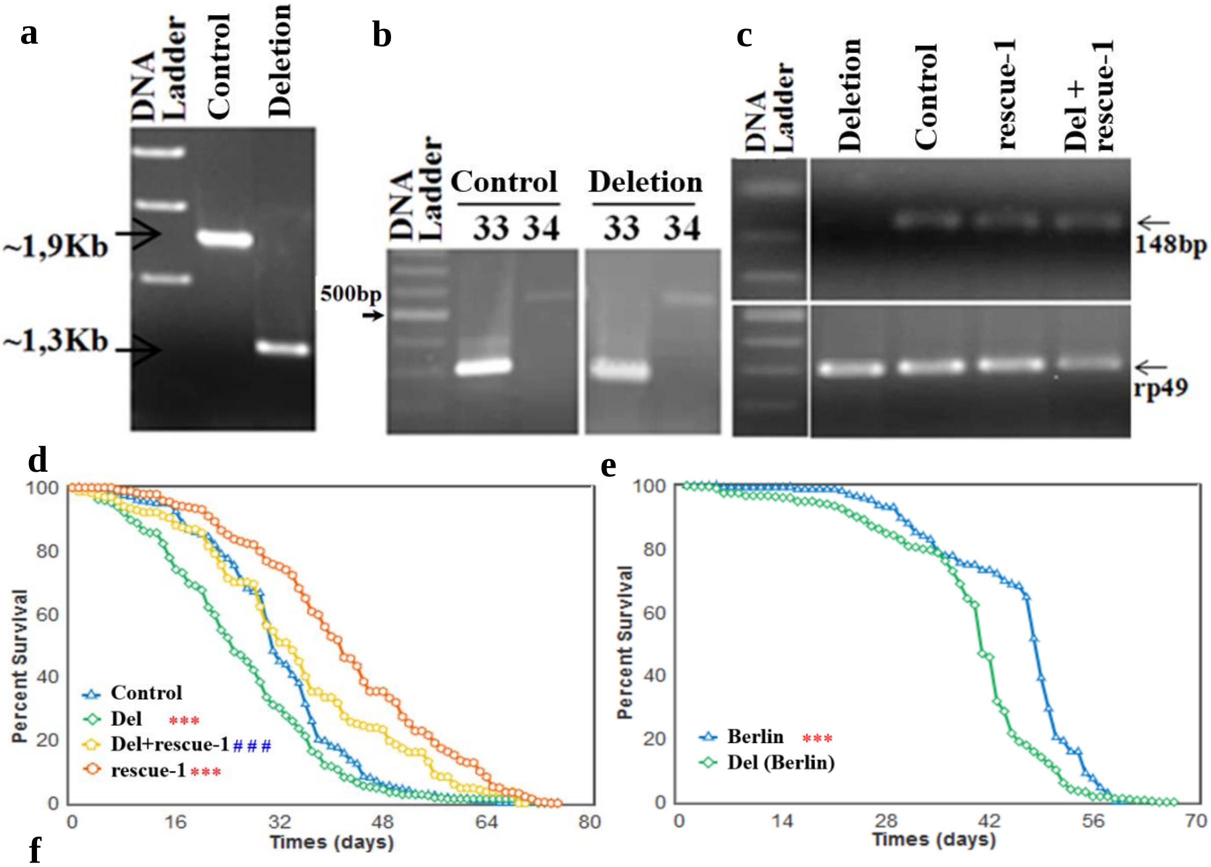

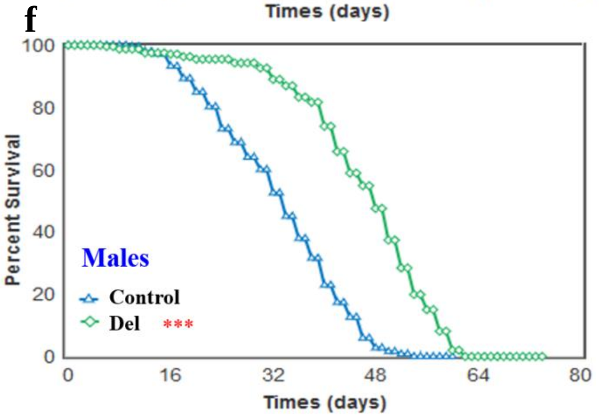

Supplementary Figure 1 | Molecular features of the snoRNA:Ψ28S-1153 (*jouvence*).

a-b-c) Agarose gel electrophoresis showing: (**a**) the deletion (F4) of 632bp, (**b**) the RT-PCR products of whole females assessing the expression of the two genes (CG13333, short-named 33) and (CG13334, short-named 34), in Control (CS) and deletion (F4) and (**c**) the expression (RT-PCR products) of the snoRNA:Ψ28S-1153 (*jouvence*). The DNA band of *jou* (148 bp) is present in control (CS), but absent in deletion. Transgenic flies without and with the deletion (rescue-1 and Del+rescue-1), also show a 148 bp band, confirming the expression of *jou*. Lower panel: rp49 used as control. (**d**) Decreasing cumulative of the second and independent experiments of females Control (CS), Del (deletion F4), Del+rescue-1 and rescue-1. (**e**) Decreasing cumulative of the females deletion in the Berlin genetic background compared to their respective controls (Co-Isogenic-Berlin). As within the Cantons-S genetic background, the deletion flies have a reduced lifespan. (**f**) Decreasing cumulative of males Control (CS), and Del (deletion F4) (for number of flies, age in days at % mortality, and detailed Statistics, see Table-S1) *** = p<0,001, compared to Control; ### = p<0,001 compared to Deletion). p-value calculated by log-rank test. For the d,e,f panels, Source data are provided as a Source Data file.

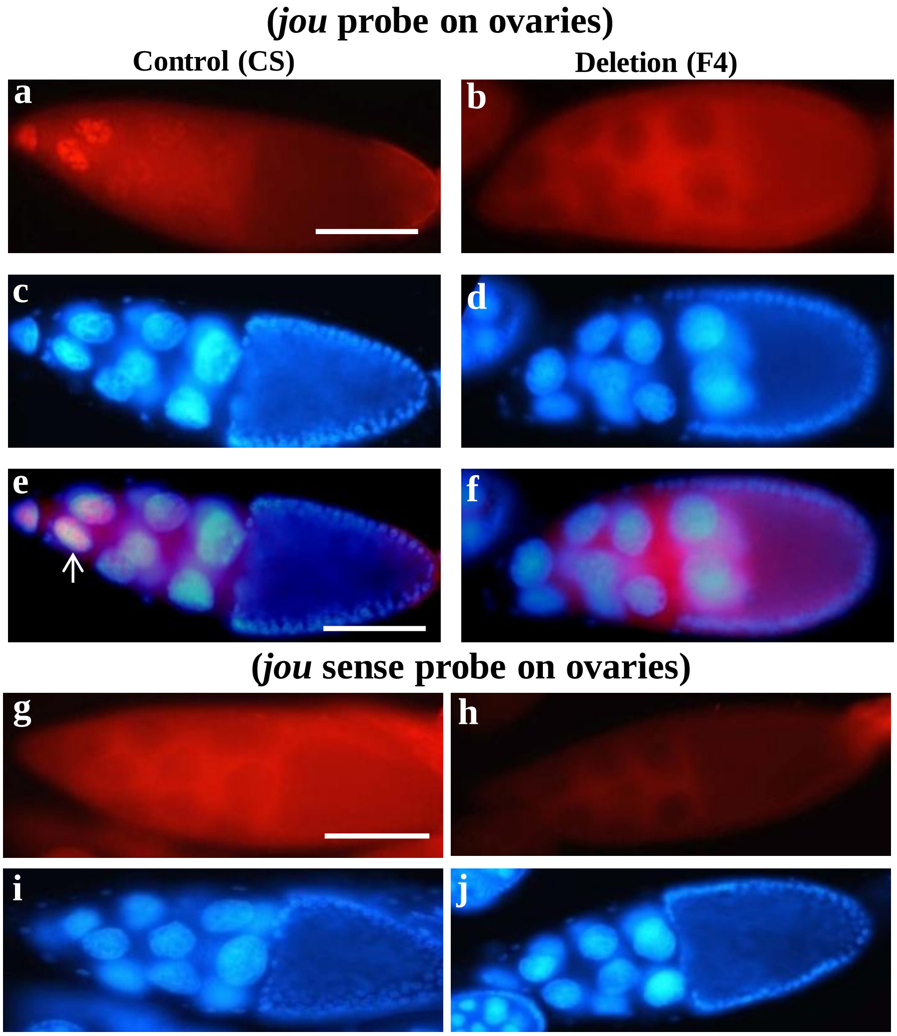

Supplementary Figure 2 | *In situ* hybridization on ovaries and *jou* sense probe used as negative control.

(**a to f**) *in situ* Hybridization (ISH) of *jou* in ovaries. (**a**) Expression in Control-CS and (**b**) in deletion flies. As expected, *jou* is not expressed in deletion. (**c-d**) Counterstaining with DAPI to label the nucleus, in Control (**c**) and deletion (**d**). (**e-f**) overlay images of (**a-c**) and (**b-d**) respectively, revealing *jou* in the nucleolus of nurse-cells (white arrow) (scale bar = 100mm). (**g to j**) ISH on ovaries using the sense probe of *jou* as negative control. Left panel: Control flies. Right panel: deletion. As expected, *jou* is not detected in either Control (**g**) or deletion (**h**). DAPI counter-staining to label the nucleolus (**i**) and (**j**) (scale bar = 100mm).

Supplementary Figure 3 Soulé et al.,

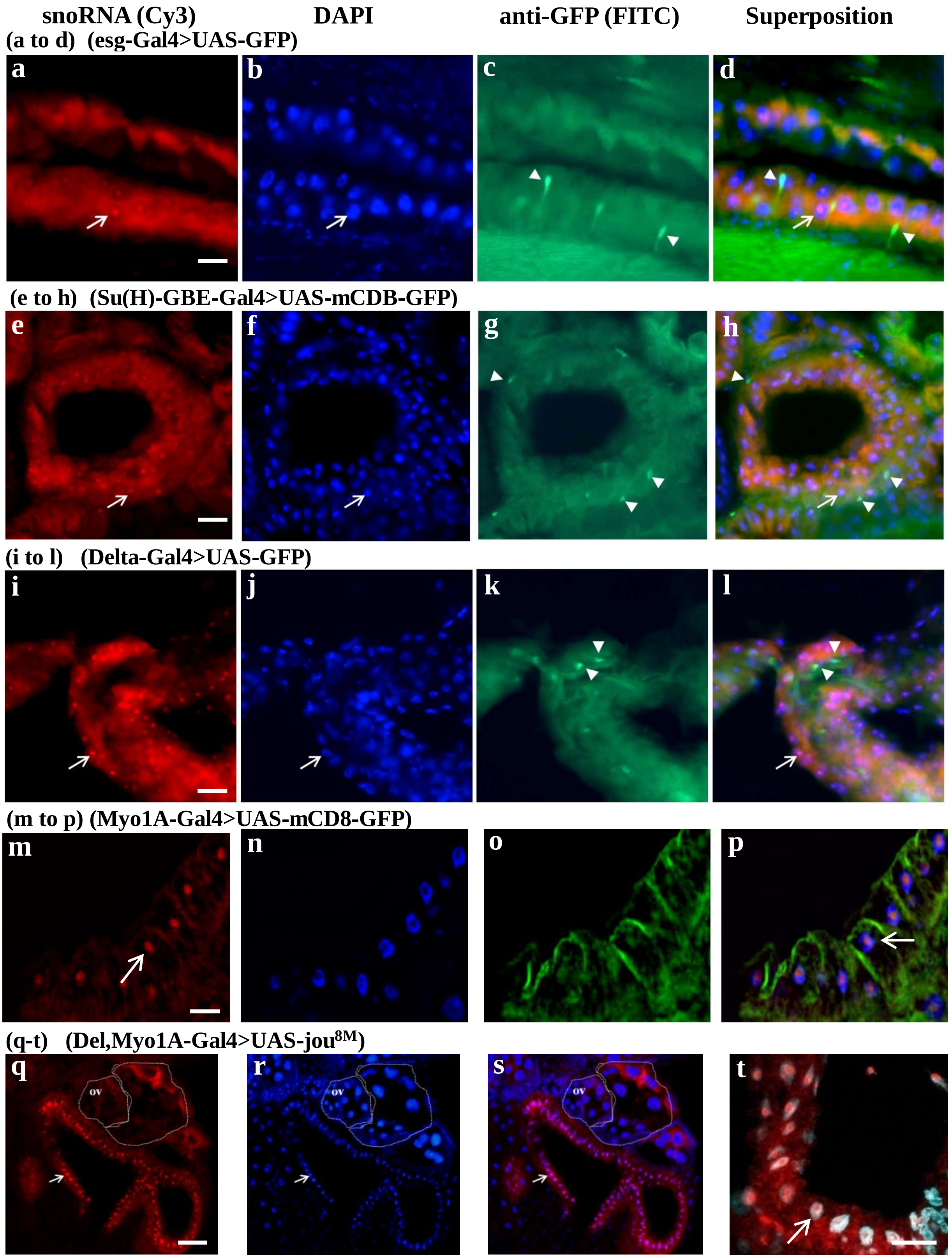

Supplementary Figure 3 | *In situ* hybridization reveals that *jou* is expressed only in enterocytes.

ISH on four different gut-Gal4 drivers demonstrate that *jou* is specifically expressed in enterocytes, and is not detected in other epithelial cell types. Left panel: *jou* snoRNA expression revealed with fluorophore tyramide-Cy3 amplification (red dots: white arrow). Second panel from the left: DAPI staining (blue). Third panel from the left: antibody staining directed against the targeted GFP (anti-GFP) (FITC in green: white arrowhead). Fourth panel from the left: Superimposed images to reveal co-localisation. Triple labelling in (**a to d**) esg-Gal4>UAS-GFP line labelling ISCs, (**e to h**) Su(H)-GBE-Gal4>UAS-mCDB-GFP labelling EBs, and (**i to l**) Delta-Gal4>UAS-GFP labelling ISCs showed that these cells in green (white arrowhead) do not express *jou* (white arrow). (**m to p**) Triple labelling of *jou* snoRNA, nucleus and GFP reporter gene in transgenic flies with targeted expression of GFP in the gut enterocytes using the Myo1A-Gal4 driver (Myo1A-Gal4>UAS-mCD8-GFP). (**m**) the *jou* expression in red (white arrow) revealed by ISH (tyramide-Cy3), (**n**) DAPI staining in blue, (**o**) anti-GFP staining in green (FITC), and (**p**) overlay of the three images revealed that *jou* is expressed exclusively in the nucleolus of enterocytes. E) Double labelling of *jou* located in the nucleus in deletion specifically expressing *jou* in enterocytes (Myo1A-Gal4>UAS-*jou*^{8M} in deletion). (**q**) *jou* expression in the epithelium of the gut (red dots: white arrow), but as expected, no expression in the ovaries (ov), (**r**) DAPI staining in blue, and (**s**) overlay of both images revealed that *jou* is expressed exclusively in the nucleolus of the enterocytes. (**t**) Magnified view taken with a confocal (63X) revealed that *jou* is located in the nucleolus. All panels: scale bar = 25mm, except (**q**) = 50mm.

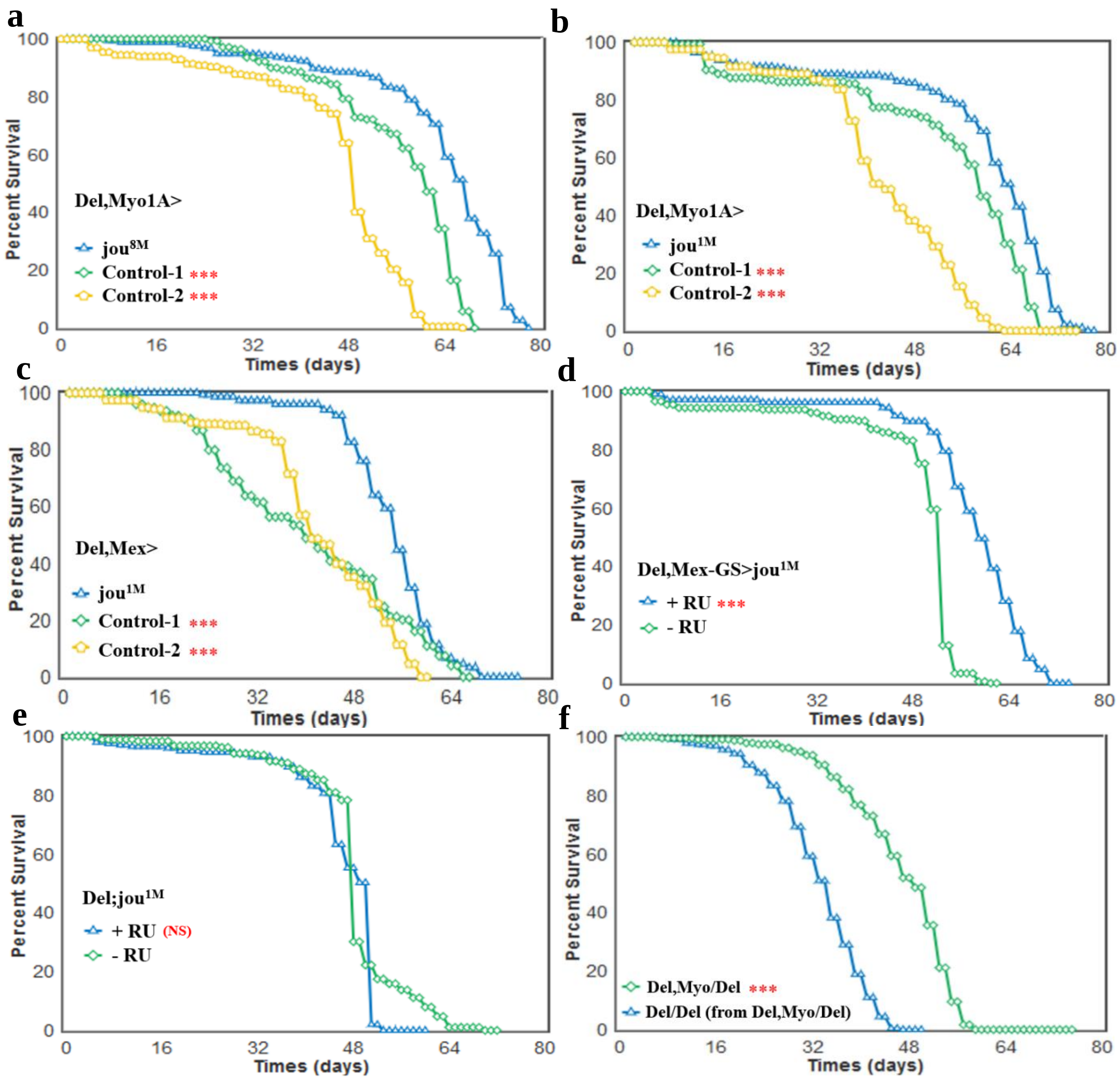

Supplementary Figure 4 | Replicates of longevity rescue experiments

Longevity test results (survival curve - decreasing cumulative) of the targeted expression of the snoRNA:Ψ28S-1153 specifically in the enterocytes in deletion compared to their controls. (**a**) Replicate of the Del,Myo1A-Gal4>UAS-jou^{8M} expression in enterocytes is sufficient to increase lifespan. (**b**) Myo1A-Gal4 driving the second transgenic flies line (pJFRC-MUH-jou^{1M} in the deletion background (Del,Myo1A-Gal4>pJFRC-MUH-jou^{1M}) in enterocytes also increases lifespan. (**c**) Mex-Gal4 driving the second transgenic flies line (pJFRC-MUH-jou^{1M} in the deletion background (Del,Mex-Gal4>pJFRC-MUH-jou^{1M}) in enterocytes also increases lifespan. (**d**) Similarly, the conditional Mex-GS driving the second transgenic flies line (pJFRC-MUH-jou^{1M}) in the deletion background (Del,Mex-GS>pJFRC-MUH-jou^{1M}) fed with the RU486 only in adulthood, which triggers the expression of the snoRNA-*jou*, also increases lifespan compared to the non-fed (non-induced) sibling flies. (**e**) RU486 control: feeding the RU486 to Del;pJFRC-MUH-jou^{1M} (without the Mex-GS driver) does not modify lifespan. (**f**) Longevity of Deletion flies (Del) recovered from the Del,Myo1A line compared to their parental line (Del,Myo-1A) reveals that the Deletion flies have similar lifespan to the original Deletion (shown in Fig. 1b and 1c), while the Del,Myo-1A shows a longer lifespan. This result demonstrates that the Myo1A enhancer-trap line has a longevity phenotype *per se* in the deletion genetic background. (for number of flies, age in days at % mortality, and detailed Statistics, see Table-S1) (*** = p<0,001). p-value calculated by log-rank test. For all panels, Source data are provided as a Source Data file.

Supplementary Figure 5

Soulé et al.,

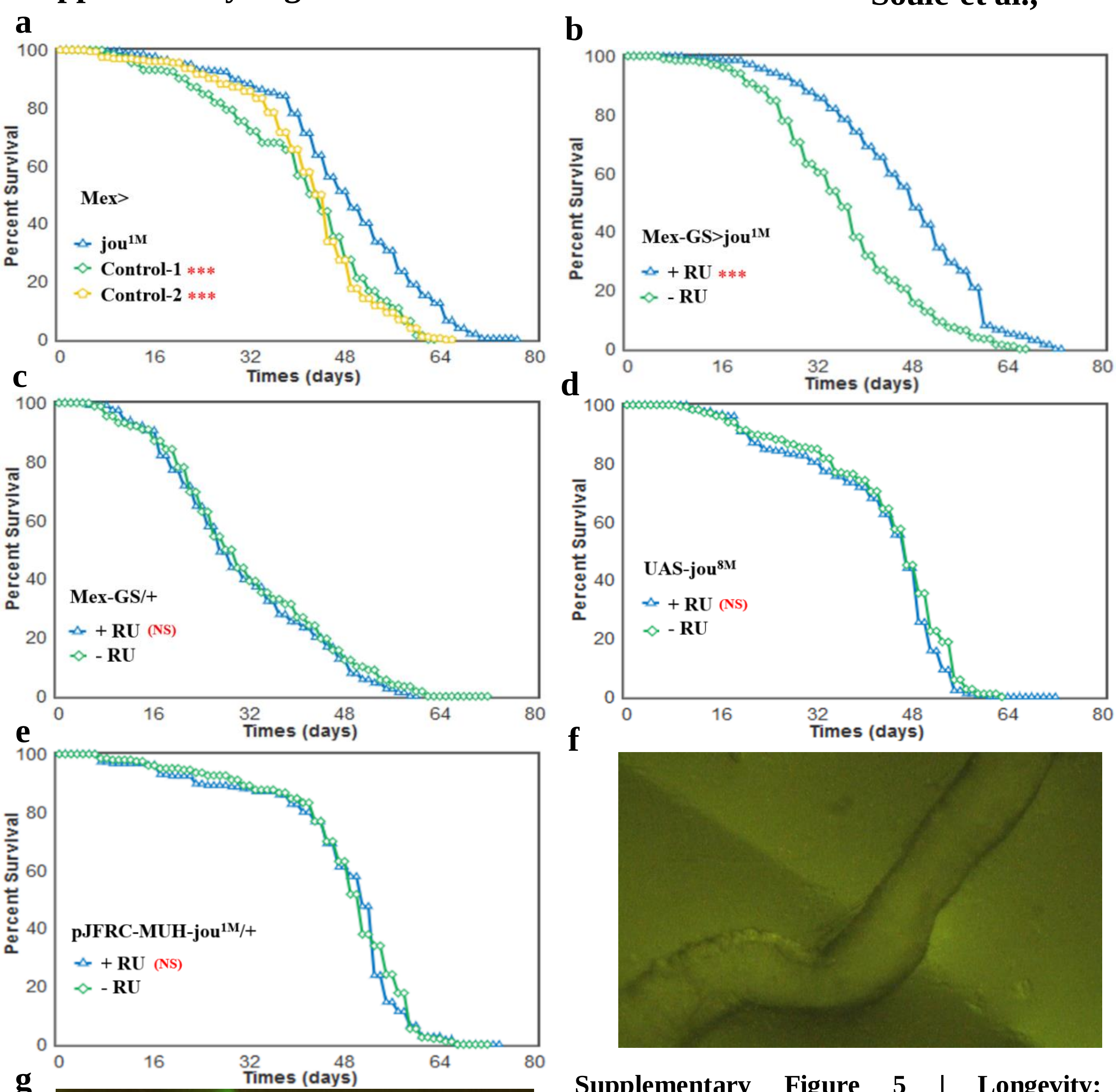

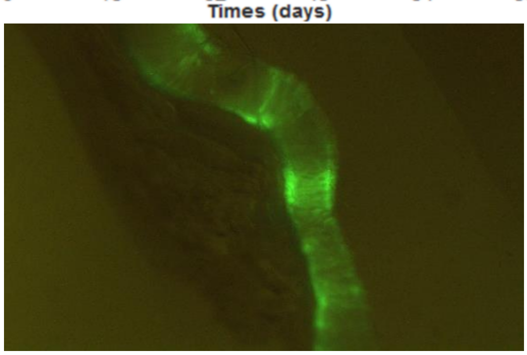

Supplementary Figure 5 | Longevity: overexpression of *jou* in enterocytes increases lifespan.

Longevity test results (survival curve - decreasing cumulative) of the targeted overexpression of *jou* specifically in the enterocytes in Wild-Type Canton-S genetic background compared to their controls. (**a**) Gal4>UAS-jou^{1M} overexpression in enterocytes also increases lifespan. (**b**) Mex-GS>UAS-jou^{1M}, with and without feeding RU486 increases lifespan. (**c-d-e**) Three different RU486 controls: feeding the RU486 to the driver line alone (Mex-GS) (without the UAS-jou construct) (**c**), or to the UAS-jou^{8M} alone (without a Gal4-driver) (**d**), or to the pJFRC-MUH-jou^{1M} alone (**e**) have no effect on lifespan. (**f-g**) Feeding the RU486 to the Mex-GS>UAS-GFP flies induces the expression of the GFP in the gut (**g**), while the GFP is not expressed in non RU486 fed flies (**f**). This results demonstrate that the Mex-GS transgenic flies are functional and expressed in the gut. p-value calculated by log-rank test. For the a,b,c,d,e panels, Source data are provided as a Source Data file.

Supplementary Figure 6 **Soulé et al.,**

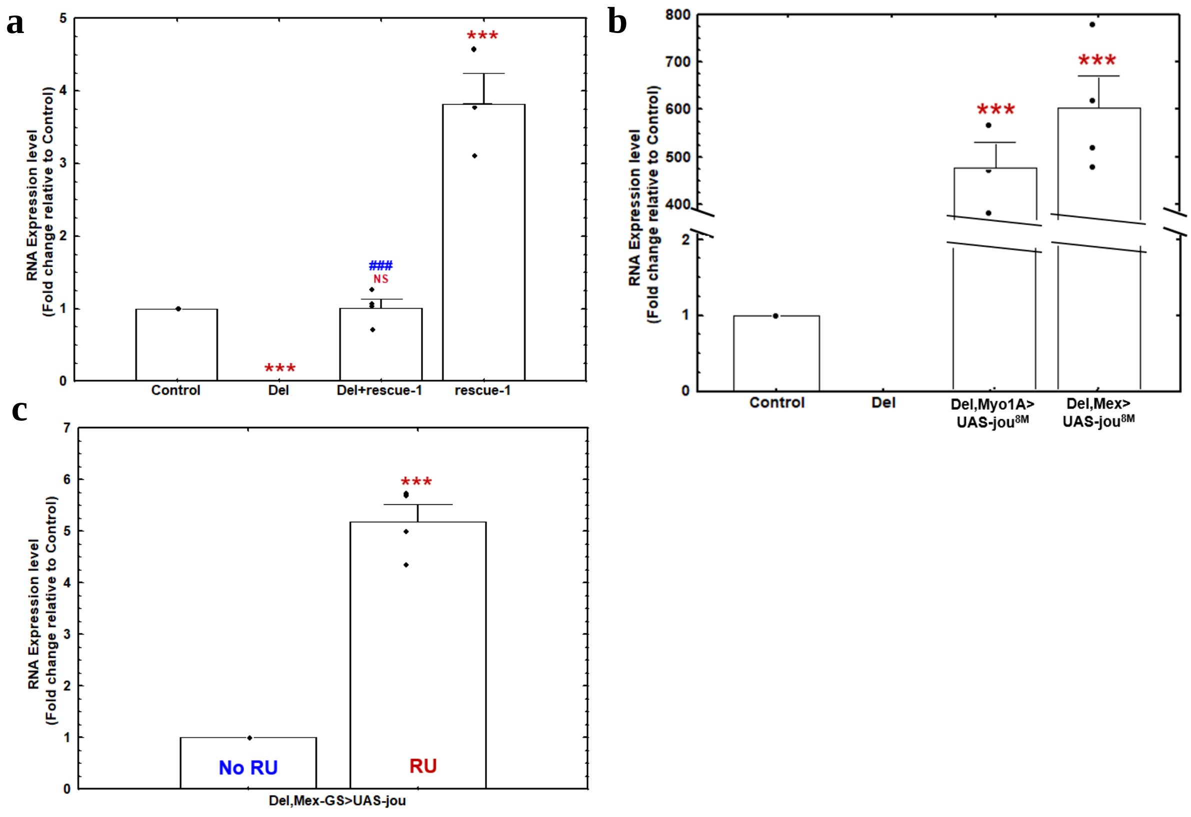

Supplementary Figure 6 | Relative RNA levels of *jou* expression from total-RNA of the gut.

Histogram of RT-qPCR (Taqman) showing the relative levels of *jou* expression from the total-RNA of the gut. (**a**) Control (CS: n=4), Del (deletion F4: n=4), genomic-rescued in the deletion (Del+rescue-1: n=4), and genomic-rescued transgene in the Wild-Type (rescue-1: n=3) (overexpression) (normalised and compared to Control-CS=1) (n=6 for each genotypes). (**b**) Quantification of the targeted expression of *jou* specifically in the enterocytes, both in Myo1A-Gal4 and Mex-Gal4 driving the UAS-jou^{8M} in the deletion background, compared to the deletion. The expression is highly increased in flies expressing *jou* (Del,Myo1A-Gal4: n=3, and Del,Mex-Gal4: n=4) (for Control and Del: same data than **a**). (**c**) The conditional Mex-GS flies (Del,Mex-GS>jou^{8M}) fed with RU486 only in adulthood confirms the induction of the expression of *jou* compared to their sibling flies without RU486 (for each condition, n=4). (*** p<0,0005 compared to CS, ### p<0,0005 compared to F4). The snoRNA-*jouvence* is normalized to the level of rp49. For each condition, 30 guts were dissected for the total RNA extraction. In all panels, error bars represent the mean +/- S.E.M. (p-value were calculated using the one-way ANOVA followed by a TUKEY test). For all panels, Source data are provided as a Source Data file.

a CS vs Deletion (Up-regulated genes)

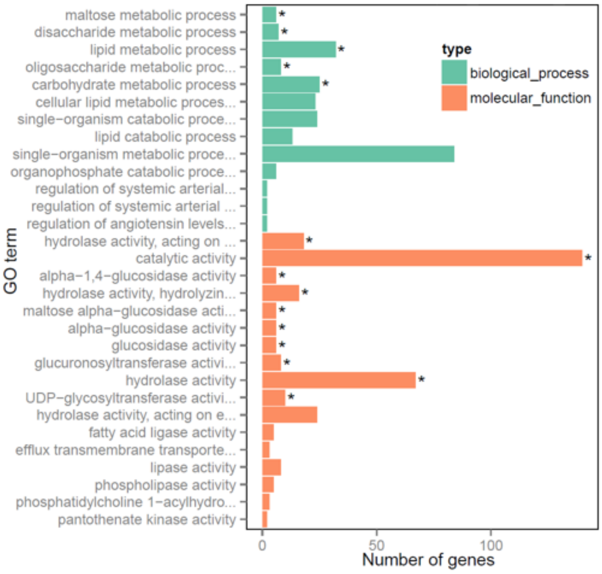

b CS vs Deletion (Down-regulated genes)

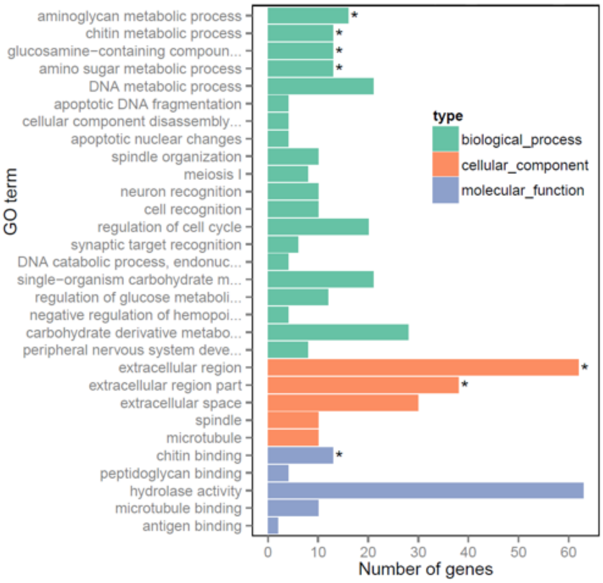

c [Mex-GS>jou^{8M} (RU vs No-RU) (Up-regulated genes)]

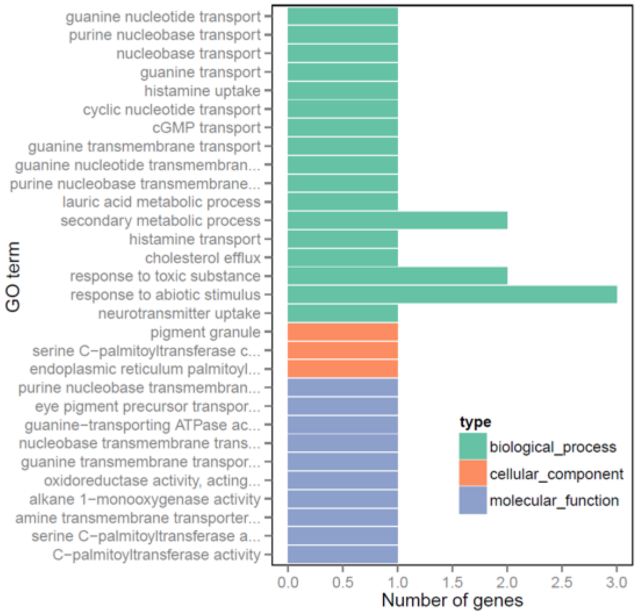

Supplementary Figure 7 | Gene Ontology Analysis (GO) of the deregulated genes.

(**a-b**) Deletion versus control-WT flies. In brief, for the up-regulated genes (**a**), the majority presents a catalytic activity, whereas, for the down-regulated genes (**b**), the majority falls in the hydrolase activity category. (**c**) Overexpression (Mex-GS>UAS-jou^{8M}) fed with RU486 versus non-RU486 fed flies. For the complete list of genes, see Suppl. Tables-S7-S8 and S11 respectively.

Supplementary Figure 8

Soulé et al.,

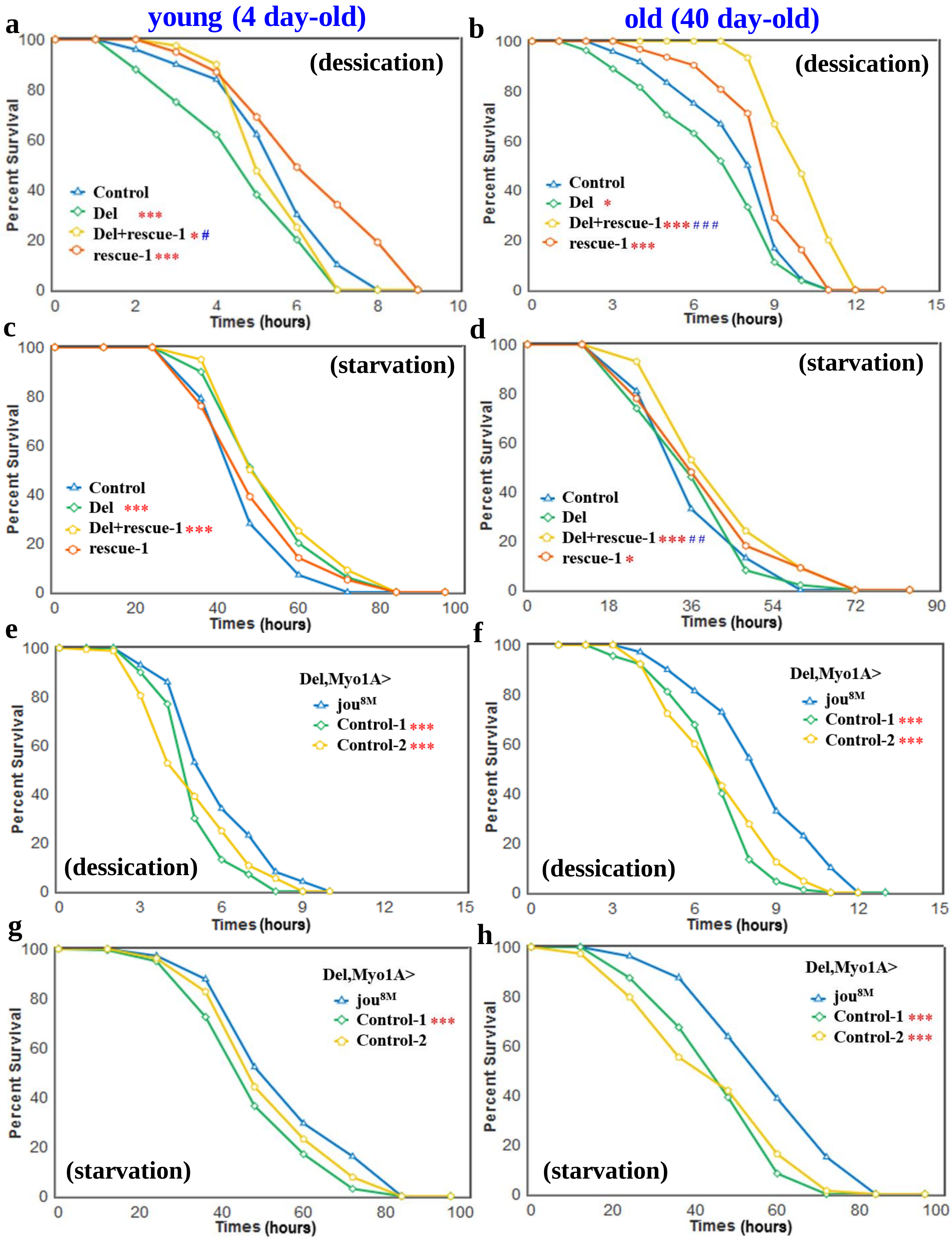

Supplementary Figure 8 | Desiccation and Starvation tests

(**a-b**) Desiccation and (**c-d**) Starvation tests in young (**a-c & e-g**) and old flies (**b-d & f-h**) of genomic-rescued flies (Del+rescue-1) (**a-b-c-d**) and of enterocytes targeted *jou* through Myo1A-Gal4 line (Del,Myo1A>UAS-jou^{8M}) (**e-f-g-h**). Both in young and old flies, the targeted expression of the snoRNA-*jou* in the enterocytes increases the resistance to desiccation and starvation, compared to their co-isogenic control flies (Del,Myo1A/Del and UAS-jou^{8M}/+) (for Statistics: see Table-S2) p-value calculated by log-rank test. For the all panels, Source data are provided as a Source Data file.

```
sno3 GTTGTCCAATGCACGCTCAACTGCCCATTTTCCAGGGTAGCAGAGTGCTATTTT-GAAGG 59
sno2 GTTGTCCCATGCACGCCCATTTGCCCATTTTCCAGGGTAATTGGGTGCTATTTTTGAAGG 60
     *******.******** **: ****************** . :*.********** *****

sno3 ACATATAGAAAACCCATCGACAATCTTACC-AAATGTCTGGCCTATTTCGTGGGCCTTGC 118
sno2 ACATATAGAAATCATGTGAACAATCTTACCGAAACGTTTTGCTAATTTTTTGG-CACTGC 119
     ***********:*. .* .*********** *** ** * ** :****  *** *. ***

sno3 ATTTCCTTTAAATTGTCAAACAATT 143
sno2 TTTTCATTTAAATTGTTGAACAATT 144
     :****.********** .*******
```

Supplementary Figure 9 | The snoRNA-2 and snoRNA-3 present a high degree of identity.

In yellow: the H-box (ANANNA) is complete in the snoRNA-3 but not in the snoRNA-2, in blue: the main difference, in green: the ACA-box.

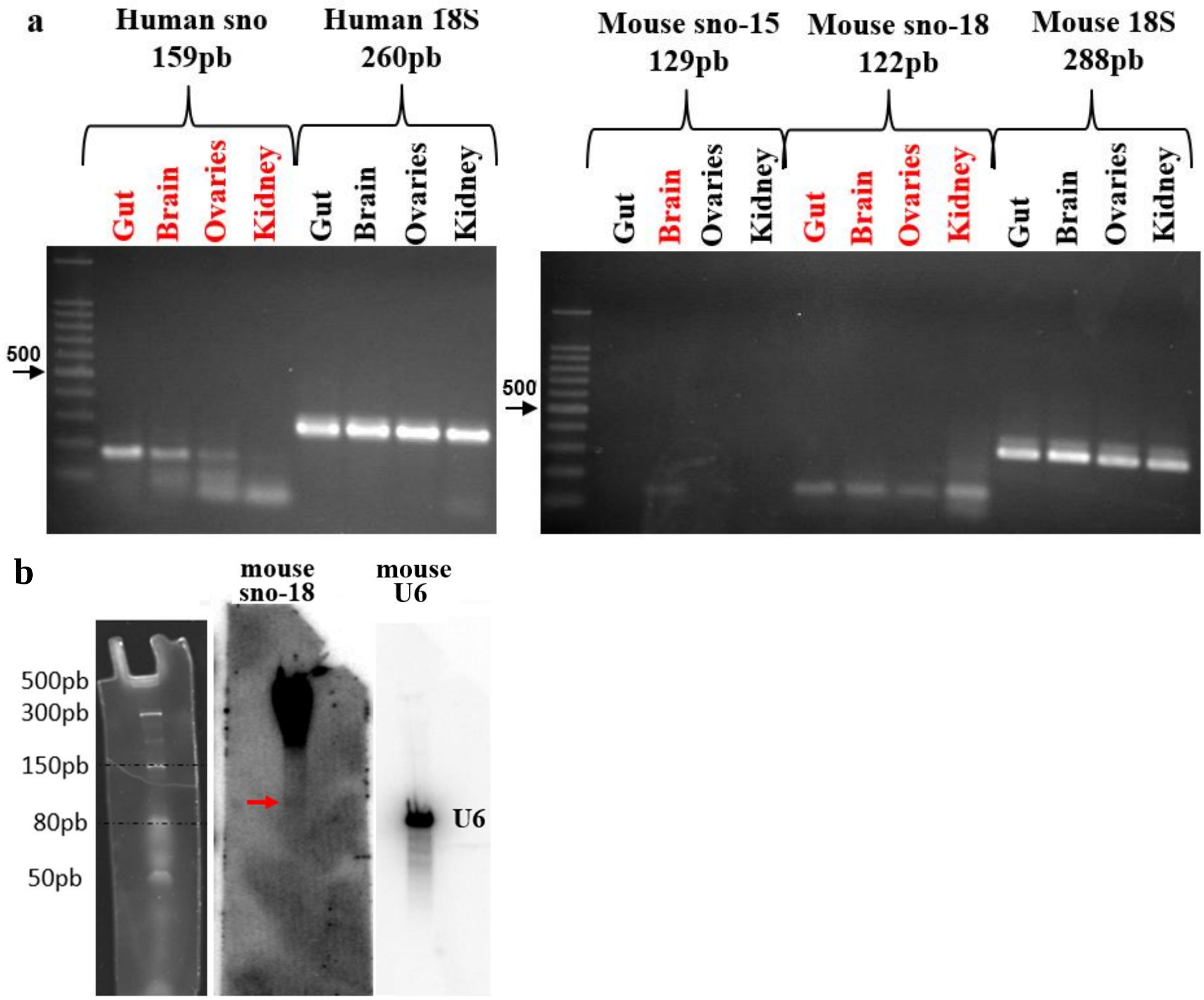

Supplementary Figure 10 | Mammalian orthologues of the snoRNA-*jouvence*.

(**a**) The RT-PCR products assessing the expression of the snoRNA in the gut, brain, ovaries and kidney of the human snoRNA (159 bp) and of the two mouse snoRNAs (snoRNA-15=129 bp) and (snoRNA-18=122 bp). In human, *jou* is detected in the gut, brain, weakly in ovaries and kidney. In mouse, the snoRNA-15 is detected in brain, but not in the gut, ovaries, and kidney. The mouse snoRNA-18 is detected in the gut, brain, ovaries, and kidney. On the right side of each gel, the human 18S and mouse 18S used as positive control, for each respective tissues. The tissues that express a snoRNA are labeled in red. (**b**) Northern Blot on total RNA extracted from the mouse gut reveals that the snoRNA-18, the one expressed in the gut, is faintly detected and processed at 122 nucleotides [notice the high level background noise due to the long exposition (7 days) to detect the signal. On the right side, the same Northern Blot re-probed with U6 (107 nucleotides) used as loading control (exposition = 2 hours)].

a **Human snoRNA: (in TEA domain family member 1 gene (SV40 transcriptional enhancer factor)**
(Location: 12,694,856..12,965,21 3Length: 270,358)

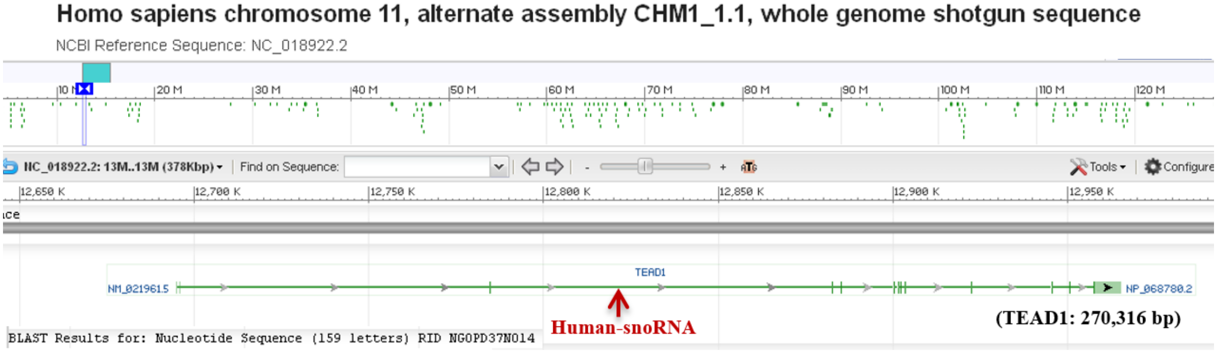

b **mouse snoRNA-15: in Ctnnd2 gene = catenin delta-2 (cadherin associated protein)**

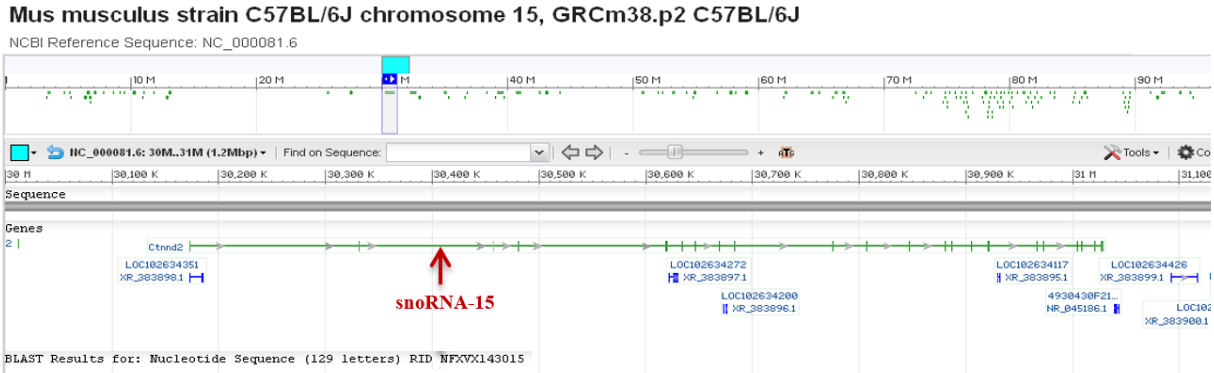

c **mouse snoRNA-18 (in Epc1 gene = Enhancer of polycomb homolog 1 (Drosophila))**

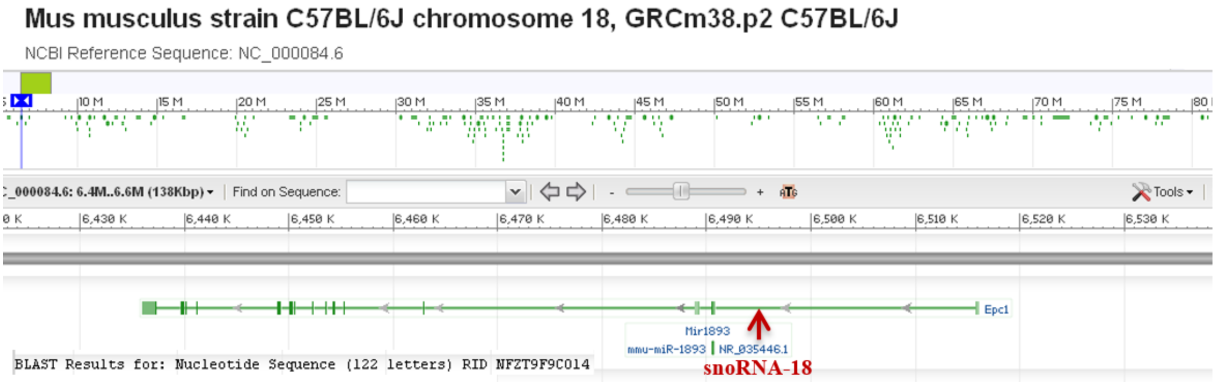

Supplementary Figure 11 | *jouvence* mammalian orthologues are located in a long intron of specific genes.

(**a**) In human, the snoRNA *jouvence* is located in a long intron of the TEAD1 (TEA domain family member 1) gene. In mouse (**b**) the snoRNA-15 is located in the long intron of the Ctnnd2 (catenin delta-2: a cadherin associated protein) gene, while (**c**) the snoRNA-18 is located in the gene EPH1 (enhancer of polycomb homolog 1 isoform 1).

Supplementary Figure 12 **Soulé et al.,**

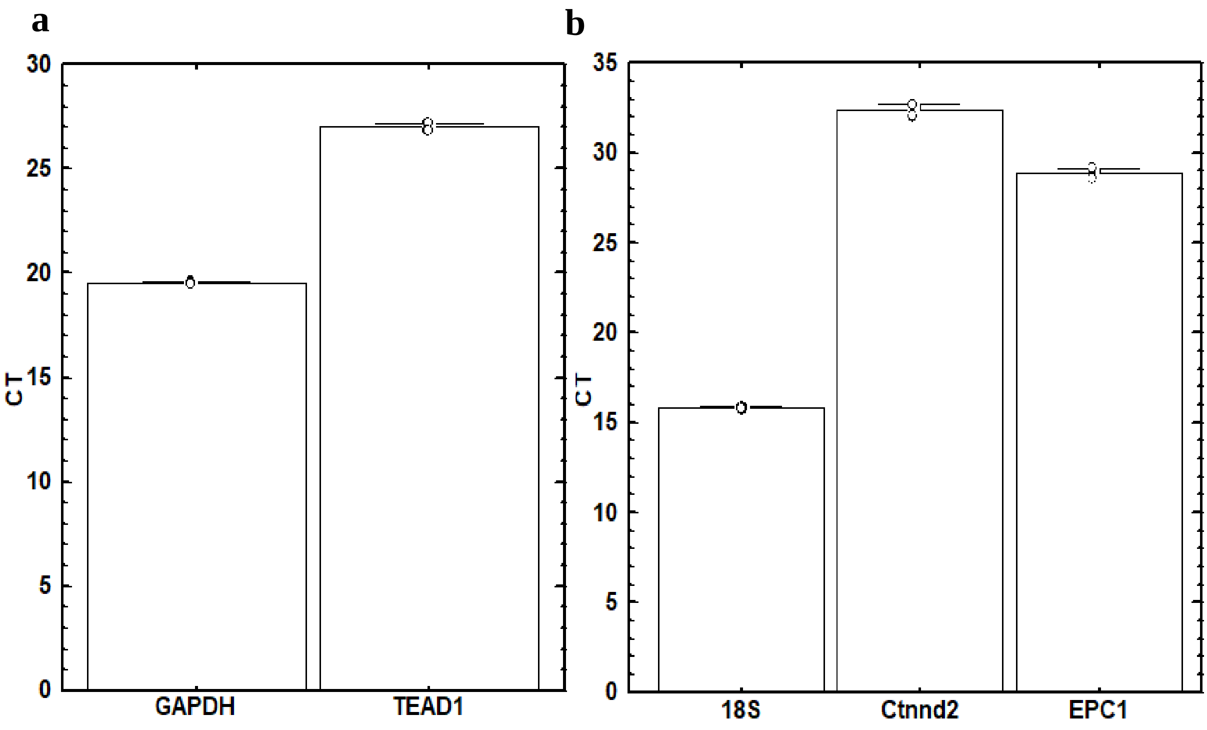

Supplementary Figure 12. The genes encompassing the snoRNA-*jouvence* are expressed in the gut.

(**a**) CT histogram of the Human TEAD1 gene revealing that this last is expressed in the gut (ΔCT=7,1) compared to the reference gene GAPDH. (**b**) CT histogram of the mouse Ctnnd2 gene (ΔCT=7,0), and EPC1 (ΔCT=13,2) revealing that these last are faintly expressed in the gut, compared to the reference gene 18S-rRNA. n=2 for each panel (gene). Error bars represent the mean +/- S.E.M. (p-value were calculated using the one-way ANOVA followed by a TUKEY test). For all panels, Source data are provided as a Source Data file.

Supplementary Figure 13 **Soulé et al.,**

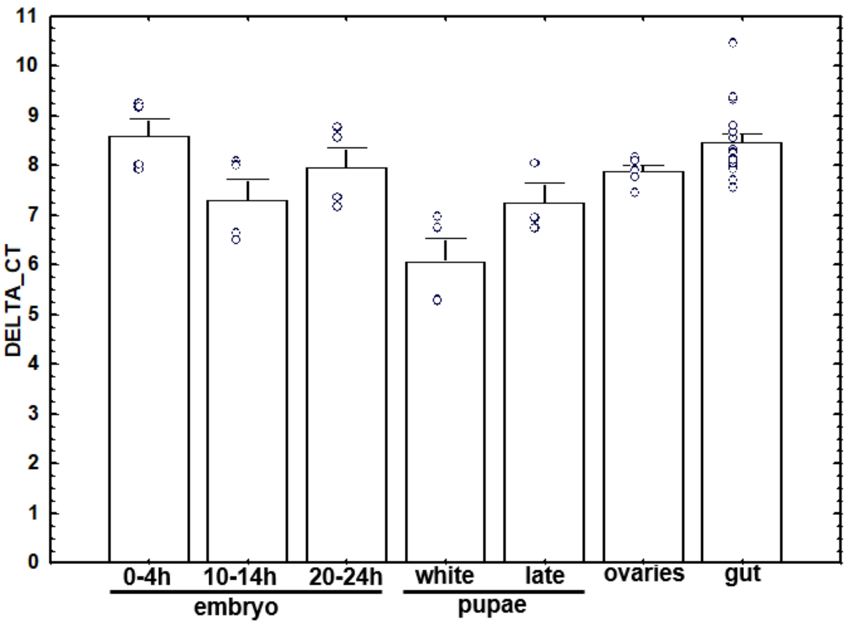

Supplementary Figure 13. Developmental expression of *jouvence* (RT-qPCR).

In *Drosophila*, RT-qPCR (Taqman) reveals that the snoRNA *jouvence* is expressed, but weakly, at different developmental stages. The results are presented as the Δ-CT compared to the reference gene rp49. These results are in accordance to the low expression level revealed by the modENCODE reported in Flybase (http://flybase.org/). Error bars represent the mean +/- S.E.M. (p-value were calculated using the one-way ANOVA followed by a TUKEY test). 0-4h, 10-14h, 20-24h, and white pupae: n=4, late pupae: n=3, ovary: n=5, gut: n=17. Source data are provided as a Source Data file.

Supplementary Figure 14 **Soulé et al.,**

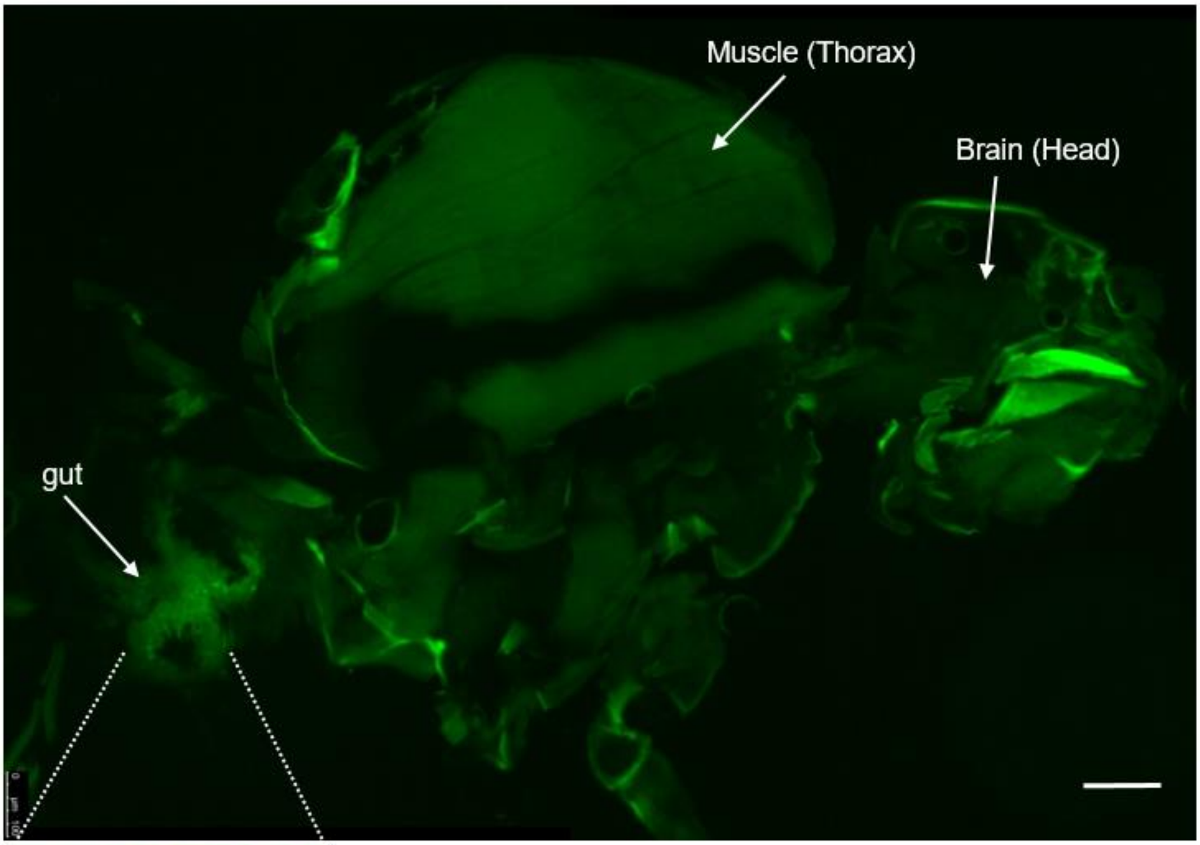

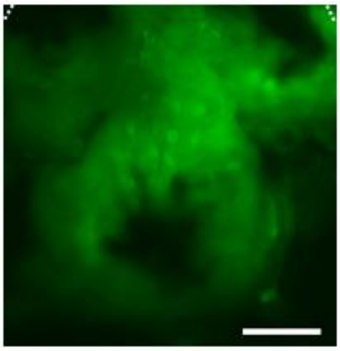

Supplementary Figure 14. Expression pattern of the Mex-GS>UAS-GFP-nls after induction by RU486.

Expression pattern of the Mex-GS>UAS-GFP-nls of a 15 day-old female fly following feeding by RU486 (since 15 days). We can remark that the GFP is expressed, as expected, only in the epithelium of the gut, but not in the muscle (thorax) or in the brain (head) or any other cells. Since we use, for visualisation, directly the fluorescence of the GPF, we can also observe some non-specific autofluorescence due to the cuticule surrounding the head and partly the thorax.

Supplementary Figure 15

Soulé et al.,

a

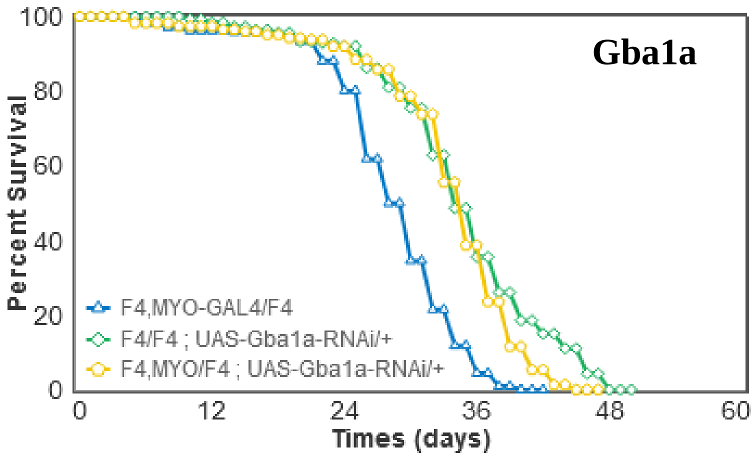

b

Name	No. of subjects	Age in days at % mortality				
		25%	50%	75%	90%	100%
F4,MYO-GAL4/F4	312	26	28	32	36	40
F4/F4 ; UAS-Gba1a-RNAi/+	253	32	34	40	46	48
F4,MYO/F4 ; UAS-Gba1a-RNAi/+	225	31	35	37	41	45

c

Condition	P-value
F4,MYO-GAL4/F4 v.s. F4/F4 ; UAS-Gba1a-RNAi/+	0
F4,MYO-GAL4/F4 v.s. F4,MYO/F4 ; UAS-Gba1a-RNAi/+	0
F4/F4 ; UAS-Gba1a-RNAi/+ v.s. F4,MYO/F4 ; UAS-Gba1a-RNAi/+	0.0017

Supplementary Figure 15 | Restoring the mRNA level of Gba1a does not rescue the lifespan.
a) Longevity test results (survival curve - decreasing cumulative) of the targeted expression of a RNAi against the Gba1a gene (Del,Myo-Gal4>UAS-Gba1a-RNAi) specifically in the enterocytes in deletion (F4) compared to their two respective controls does not rescue the lifespan of the flies, and even it is slightly deleterious when compared to one control line. B) Numbers of female flies for each genotype, and age in days at % mortality. C) Statistics: calculated by log-rank test. Source data are provided as a Source Data file.

Description of Additional Supplementary Files

(RNA-Seq Analysis)

Supplementary Data 1: Deletion versus Control (Wild-Type)
Differentially expressed upregulated genes (sorted-p value)

Supplementary Data 2: Deletion versus Control (Wild-Type)
Differentially expressed down-regulated genes (sorted-p value)

Supplementary Data 3: Deletion versus Control (Wild-Type)
Differentially expressed upregulated genes: KEGG pathway

Supplementary Data 4: Deletion versus Control (Wild-Type)
Differentially expressed down-regulated genes: KEGG pathway

Supplementary Data 5: Deletion versus Control (Wild-Type)
Differentially expressed upregulated genes: GO enrichment

Supplementary Data 6: Deletion versus Control (Wild-Type)
Differentially expressed down-regulated genes: GO enrichment

Supplementary Data 7: MexGS>UAS-jou^{8M} (RU) versus MexGS-Control (No RU)
Differentially expressed upregulated genes (sorted-p value)

Supplementary Data 8: MexGS>UAS-jou^{8M} (RU) versus MexGS-Control (No RU)
Differentially expressed upregulated genes: KEGG pathway

Supplementary Data 9: MexGS>UAS-jou^{8M} (RU) versus MexGS-Control (No RU)
Differentially expressed upregulated genes: GO enrichment
