# Supplementary material for: Jouvence a small nucleolar RNA required in the gut extends lifespan in Drosophila
Source: Nat Commun. 2020 Feb 20;11:987. doi: 10.1038/s41467-020-14784-1 (PMC7033134; doi:10.1038/s41467-020-14784-1)
Supplement: Supplementary file 3 — Description of Additional Supplementary Files [file 41467_2020_14784_MOESM3_ESM.docx]

**Description of Supplementary Files**

**File Name: Supplementary Data 1**

**Description:** Deletion versus Control (Wild-Type). Differentially expressed upregulated genes (sorted-p value).

**File Name: Supplementary Data 2**

**Description:** Deletion versus Control (Wild-Type). Differentially expressed down-regulated genes (sorted-p value).

**File Name: Supplementary Data 3**

**Description:** Deletion versus Control (Wild-Type). Differentially expressed upregulated genes: KEGG pathway.

**File Name: Supplementary Data 4**

**Description:** Deletion versus Control (Wild-Type). Differentially expressed down-regulated genes: KEGG pathway.

**File Name: Supplementary Data 5**

**Description:** Deletion versus Control (Wild-Type). Differentially expressed upregulated genes: GO enrichment.

**File Name: Supplementary Data 6**

**Description:** Deletion versus Control (Wild-Type). Differentially expressed down-regulated genes: GO enrichment.

**File Name: Supplementary Data 7**

**Description:** MexGS>UAS-jou8M (RU) versus MexGS-Control (No RU). Differentially expressed upregulated genes (sorted-p value).

**File Name: Supplementary Data 8**

**Description:** MexGS>UAS-jou8M (RU) versus MexGS-Control (No RU). Differentially expressed upregulated genes: KEGG pathway.

**File Name: Supplementary Data 9**

**Description:** MexGS>UAS-jou8M (RU) versus MexGS-Control (No RU). Differentially expressed upregulated genes: GO enrichment.
