# Supplementary material for: Jouvence a small nucleolar RNA required in the gut extends lifespan in Drosophila
Source: Nat Commun. 2020 Feb 20;11:987. doi: 10.1038/s41467-020-14784-1 (PMC7033134; doi:10.1038/s41467-020-14784-1)
Supplement: Supplementary file 13 — Reporting Summary [file 41467_2020_14784_MOESM13_ESM.pdf]

nature research

Corresponding author(s): Jean-René MARTIN

Last updated by author(s): Dec 14, 2019

Reporting Summary

Nature Research wishes to improve the reproducibility of the work that we publish. This form provides structure for consistency and transparency in reporting. For further information on Nature Research policies, see Authors & Referees and the Editorial Policy Checklist .

Statistics

For all statistical analyses, confirm that the following items are present in the figure legend, table legend, main text, or Methods section.

n/a	Confirmed	
☐	☒	The exact sample size (*n*) for each experimental group/condition, given as a discrete number and unit of measurement
☐	☒	A statement on whether measurements were taken from distinct samples or whether the same sample was measured repeatedly
☐	☒	The statistical test(s) used AND whether they are one- or two-sided<br>*Only common tests should be described solely by name; describe more complex techniques in the Methods section.*
☒	☐	A description of all covariates tested
☐	☒	A description of any assumptions or corrections, such as tests of normality and adjustment for multiple comparisons
☐	☒	A full description of the statistical parameters including central tendency (e.g. means) or other basic estimates (e.g. regression coefficient) AND variation (e.g. standard deviation) or associated estimates of uncertainty (e.g. confidence intervals)
☐	☒	For null hypothesis testing, the test statistic (e.g. *F*, *t*, *r*) with confidence intervals, effect sizes, degrees of freedom and *P* value noted<br>*Give P values as exact values whenever suitable.*
☒	☐	For Bayesian analysis, information on the choice of priors and Markov chain Monte Carlo settings
☒	☐	For hierarchical and complex designs, identification of the appropriate level for tests and full reporting of outcomes
☒	☐	Estimates of effect sizes (e.g. Cohen's *d*, Pearson's *r*), indicating how they were calculated

Our web collection on statistics for biologists contains articles on many of the points above.

Software and code

Policy information about availability of computer code

Data collection	n/a
Data analysis	For the longevity and stress resistance survival curves, log-ranks test was performed using the freely available OASIS software (https://sbi.postech.ac.kr/oasis2/). For the qPCR, and gut cells count, data were analysed statistically using analysis of variance (one-way ANOVA tests followed by a TUKEY test), with StatisticaTM software.

For manuscripts utilizing custom algorithms or software that are central to the research but not yet described in published literature, software must be made available to editors/reviewers. We strongly encourage code deposition in a community repository (e.g. GitHub). See the Nature Research guidelines for submitting code & software for further information.

Data

Policy information about availability of data

All manuscripts must include a data availability statement. This statement should provide the following information, where applicable:

- Accession codes, unique identifiers, or web links for publicly available datasets
- A list of figures that have associated raw data
- A description of any restrictions on data availability

We declare that all the data and the methods used in this study are available within this article, its Supplementary Information files, the peer-review file, or are available from the corresponding author upon reasonable request. Accession number of RNA-Seq Data at NCBI are included in the manuscript.

Field-specific reporting

Please select the one below that is the best fit for your research. If you are not sure, read the appropriate sections before making your selection.

☒ Life sciences ☐ Behavioural & social sciences ☐ Ecological, evolutionary & environmental sciences

For a reference copy of the document with all sections, see nature.com/documents/nr-reporting-summary-flat.pdf

Life sciences study design

All studies must disclose on these points even when the disclosure is negative.

Sample size	Sample sizes (for cells count/gut and Flies for longevity and stress tests) were initially determined empirically, but we verified that the power of the statistical tests used were greater that 80 % for each representative set of experiments.
Data exclusions	No data were excluded from the analysis.
Replication	Replication were extensive throughout the manuscript, as stated in Methods. Key experimental results (as longevity) were repeated independently at least twice, and for several of them, the replicates are shown in Supplementary Informations.
Randomization	Flies for all experiments were subjected to randomization of genotype and treatment.
Blinding	We were not blinded to group allocation. Since each experimental group consists of a distinct genotype, we did not allocate flies of a certain genotype into multiple groups in this study. Accordingly, we do not think that we need to be blind in this step.

Reporting for specific materials, systems and methods

We require information from authors about some types of materials, experimental systems and methods used in many studies. Here, indicate whether each material, system or method listed is relevant to your study. If you are not sure if a list item applies to your research, read the appropriate section before selecting a response.

Materials & experimental systems

n/a	Involved in the study	
☐	☒	Antibodies
☒	☐	Eukaryotic cell lines
☒	☐	Palaeontology
☐	☒	Animals and other organisms
☒	☐	Human research participants
☒	☐	Clinical data

Methods

n/a	Involved in the study	
☒	☐	ChIP-seq
☒	☐	Flow cytometry
☒	☐	MRI-based neuroimaging

Antibodies

Antibodies used	For the anti-phospho Histone-H3 (anti-PH3) immunostaining, we used polyclonal anti-rabbit-phospho-Histone-3 at 1/2000 (EMD-Milipore, #06-570), Lot # 2517793.
Validation	This primary antibody is commercial and therefore previously validated in several publications cited in the manuscript.

Animals and other organisms

Policy information about studies involving animals; ARRIVE guidelines recommended for reporting animal research

Laboratory animals	The laboratory stock w1118 Canton-S Drosophila melanogaster flies were used as reference flies. All genotypes were backcrossed at least 6 fold with this reference genetic background (isogenisation). Unless specified, females were used. For the dissected gut (cells count), young flies were 7 day-old, while old flies were 40 day-old.
Wild animals	n/a
Field-collected samples	n/a
Ethics oversight	n/a

Note that full information on the approval of the study protocol must also be provided in the manuscript.
